# Tolerated drugs in subjects with severe cutaneous adverse reactions (SCARs) induced by anticonvulsants and review of the literature

**DOI:** 10.1186/s12948-017-0072-5

**Published:** 2017-10-04

**Authors:** Fabrizio De Luca, Laura Michelina Losappio, Corrado Mirone, Jan Walter Schroeder, Antonella Citterio, Maria Gloria Aversano, Joseph Scibilia, Elide Anna Pastorello

**Affiliations:** 1 Department of Allergology and Immunology, Ospedale Metropolitano Niguarda Ca’ Granda, Piazza Ospedale Maggiore, 3, 20162 Milan, Italy; 2 Department of Burn/Intensive Care, Ospedale Metropolitano Niguarda Ca’ Granda, Milan, Italy

**Keywords:** Anticonvulsants, Drug hypersensitivity, Anticonvulsant hypersensitivity syndrome, Stevens–Johnson syndrome, Toxic epidermal necrolysis, Drug reaction with eosinophilia and systemic symptoms, Severe cutaneous adverse reactions

## Abstract

**Background:**

Anticonvulsant hypersensitivity syndrome represents a rare but potentially fatal kind of adverse drug reaction. This clinical picture often hampers the flexibility with which alternative anticonvulsants or even other classes of drugs are prescribed in these patients, negatively affecting the efficacy of treatment and the course of the disease. The aim of this study was to analyse a group of six patients with severe cutaneous drug reactions induced by anticonvulsants and to report which alternative antiepileptic drugs and which drugs of other classes were tolerated.

**Case presentation:**

A total of six patients (2 males and 4 females, age 11–73 years) are described in this study. In all the patients the onset of the severe cutaneous drug reactions was 2–4 weeks after initiating the anticonvulsant therapy: 2 out of 6 patients presented with a drug reaction with eosinophilia and systemic symptoms under therapy with phenytoin; 2 out of 6 presented with Stevens–Johnson syndrome under therapy with lamotrigine; and 2 out of 6 presented with a toxic epidermal necrolysis, one of them under therapy with valproic acid, and the other one under therapy with lamotrigine. Alternative anticonvulsants tolerated after the reaction were: clonazepam, levetiracetam, diazepam, delorazepam and lormetazepam.

**Conclusions:**

In our cases we observed that non aromatic anticonvulsants and benzodiazepines were well tolerated as alternative treatments in six patients with reactions to aromatic anticonvulsivants and that the risk of hypersensitivity reactions to other drug classes was not increased as compared to general population.

## Background

Anticonvulsant hypersensitivity syndrome (AHS) represents a rare but potentially fatal kind of adverse drug reaction. The antiepileptic drugs (AEDs) most commonly involved are the aromatic anticonvulsants such as phenytoin, phenobarbital, carbamazepine, and lamotrigine; however, in the literature several cases induced by valproic acid are also reported [1–3]. Clinical presentations are highly variable and include either simple pruritic eruptions or severe forms such as Stevens–Johnson syndrome (SJS), toxic epidermal necrolysis (TEN) and drug reaction with eosinophilia and systemic symptoms (DRESS) [4, 5]. SJS and TEN are characterized by detachment of the epidermis and erosions of mucous membranes and are considered to be the same disease, the only difference being the extent of skin detachment, < 10% of total body surface area in SJS, and > 30% in TEN [6]. DRESS is characterized by eosinophilia, skin rash, fever, lymphadenopathy, and visceral organ involvement [7]. The clinical symptoms usually develop from 1 to 8 weeks after starting the antiepileptic therapy; adults older than 64 years are at the highest risk for severe cutaneous adverse reactions (SCARs) [8]. The estimated incidence of mucocutaneous severe reactions to AEDs with internal organ involvement ranges from 1 in 1000 to 1 in 10,000 drug exposures [9]. In SJS/TEN necrosis of the skin and mucous membranes characterizes the disease; apoptosis of keratinocytes is mediated by the Fas–FasL interaction or through cytotoxic T-cell release of granulysin [10]. A genetic background confers an increased risk to develop severe forms of AHS in some ethnic groups, as demonstrated by several associations between HLA-A and -B haplotypes and severe anticonvulsant reactions, as reported in Table 1 [11–16].

**Table 1:** Associations between HLA-A and -B haplotypes and severe anticonvulsant reactions

Disease	Culprit drug	HLA haplotype	Population	Authors (ref. number)
SJS ⁄TEN	Carbamazepine	HLA-B*15:02	Asians	[11–13]
SJS/TEN and DRESS	Carbamazepine	HLA-A*31:01	Europeans	[14, 15]
SJS/TEN and DRESS	Phenytoin	HLA-B*15:13 and -B*15:02	Malaysians	[16]

SJS: Stevens Johnson syndrome, TEN: toxic epidermal necrolysis, DRESS: drug reaction with eosinophilia and systemic symptoms

As it has been demonstrated in the last decade, the T cell activation at the basis of SCARs is explainable by the so called “p-i mechanism” that does not imply any processing or metabolism of the drug, making thus reasonable a definite HLA restriction [17].

Infections play an important role in the pathogenesis of these reactions through an aspecific activation of immunological response, so that, for example the human herpes virus 6 (HHV-6) reactivation in DRESS is considered a relevant diagnostic marker [18]. Moreover, viral infections as well as other cell damaging events such surgery or severe cardiac diseases, represent the so called ‘danger’ factors able to prepare the pathogenetic background favouring the appearance of SCARs. All these aspects are crucial points to address in order to evaluate the risk of hypersensitivity reactions to alternative anticonvulsants or other types of drug. However, more studies will be necessary to better define the kind of genetic/environmental interaction that are at the basis of these syndromes. As a result, SCARs often restrict the use of alternative anticonvulsants or of other classes of drugs that have to be prescribed in these patients, negatively affecting the efficacy of treatment and the course of the disease.

The aim of this study was to describe a group of six patients with SCARs induced by anticonvulsant drugs and to report which alternative antiepileptic drugs and drugs of other classes were tolerated.

## Case reports

A total of 6 caucasian patients (2 males and 4 females, age 11–73 years), admitted to Niguarda Ca’ Granda Metropolitan Hospital for SCARs, are described in this study. In all of the patients the onset of the SCARs was 2–4 weeks after initiating the anticonvulsant therapy. For each patient we collected data regarding medical and pharmacological history. They were evaluated according to the diagnostic criteria based on RegiSCAR (Registry for Serious Cutaneous Reactions) classification (i.e. both clinical and histological features). In order to identify the inducing drug, we obtained a detailed and thorough medication history. Furthermore, a standard set of laboratory tests was performed including blood count, renal function, liver function, inflammatory markers, lactate dehydrogenase (LDH). Moreover, samples for antinuclear antibodies (ANA), and immunoglobulins against HHV-6, hepatitis virus B (HBV) and C (HCV), herpes simplex virus (HSV) type 1 and 2, human immunodeficiency virus (HIV), Epstein–Barr virus (EBV), and cytomegalovirus (CMV), blood cultures and urine cultures, were collected in order to exclude other diseases. Among the six cases, we found two cases of DRESS, two cases of SJS, and two cases of TEN. Tables 2 and 3 summarize the clinical characteristics of the study patients. Table 4 shows treatment, culprit and tolerated drugs after and during SCARs.

**Table 2:** Demographics and risk factors

Patient	Sex/age	Risk factors
P1	M/11	Surgery treatment of cerebral cancer
P2	F/32	Surgery for rupture of middle cerebral artery aneurysm
P3	M/73	Severe cachexia
P4	F/53	Acute disseminated encephalomyelitis
P5	F/41	Head trauma
P6	F/21	None

**Table 3:** Clinical and laboratory data at the diagnosis

	P1	P2	P3	P4	P5	P6
Eosinophils (%)	12	39	5.1	4.6	2.3	2.2
Leukocytes (10^9^/L)	34.67	40.23	15.0	16.0	5.6	4.7
Atypical lymphocytes	+	+	−	−	−	−
GOT/GPT (IU/L)	1034/906	1068/960	56/50	89/63	510/702	503/731
CRP (mg/dL)	9.2	9.6	13.6	11.3	1.1	1.0
LDH (IU/L)	1780	1890	1230	1360	1450	423
HHV-6 infection	+	+	−	−	−	−
HCV, HBV, CMV, HSV-1 and -2, EBV, HIV infection	−	−	HSV-2	−	−	
Blood cultures	−	−	Candida albicans	MRSA	Staphylococcus capitis	MRSA
Urine cultures	−	−	−	−	Enterococcus faecalis	−
ANA	−	−	−	−	−	−
Skin biopsy	Acantholytic cells, skin detachment, mild lymphocytic infiltration	ND	ND	Apoptotic keratinocytes, spongiosis, subepidermal blister and dermal inflammatory infiltrate	Dermal infiltrate of lymphocytes and monocytes	Apoptotic keratinocytes, epidermal necrosis and dermal infiltrate of lymphocytes

CRP: C-reactive protein, GOT: glutamate oxaloacètique transaminase, GPT: glutamane pyruvate transaminase, LDH: lactate dehydrogenase, ANA: antinuclear antibodies, MRSA: methicillin-resistant *Staphylococcus aureus*, *ND*: not done, +: positive, −: negative

**Table 4:** Treatment, culprit and tolerated drugs after and during SCARs

Patient	SCARs	Culprit drug	Latency (weeks)	Treatment	Tolerated anticonvulsants	Other tolerated drugs
P1	DRESS	Phenytoin	3	Methylprednisolone for 3 months	Clonazepam	Ibuprofen, clarithromycin
P2	DRESS	Phenytoin	3	Prednisone for 1 year	Levetiracetam	Metamizole, tramadol, amoxicillin, clarithromycin, ketoprofen, metoclopramide, rituximab, lercanidipine
P3	SJS	Lamotrigine	4	Hydrocortisone bolus, prednisone for 1 month and immunoglobulin intravenously	Delorazepam and lormetazepam	Echinocandin
P4	SJS	Phenytoin	4	Hydrocortisone bolus, prednisone for 1 month and immunoglobulin intravenously	Diazepam	Teicoplanin, haloperidol
P5	TEN	Valproic acid	2	Prednisone for 1 month and immunoglobulin intravenously	Diazepam and levetiracetam	Meropenem
P6	TEN	Lamotrigine	2	Prednisone for 1 month and immunoglobulin intravenously	Diazepam and levetiracetam	Teicoplanin

SCARs: severe cutaneous adverse reactions, SJS: Stevens–Johnson syndrome, DRESS: drug reaction with eosinophilia and systemic symptoms, TEN: toxic epidermal necrolysis

### Cases of DRESS

Two patients, an 11-year old male (P1) and a 32-year old female (P2) presented SCARs and relevant eosinophilia after 3 weeks of therapy with phenytoin, prescribed as prophylactic anticonvulsant for surgical removal of glioblastoma in P1 and neurosurgery due to the rupture of a middle cerebral artery aneurysm in P2. Both patients developed generalized papular rash, fever, asthenia, lymphadenopathy, leukocytosis, eosinophilia, and signs of liver damage, as reported in Table 3. Eosinophilia, lymphadenopathy, skin biopsy in P1, and HHV-6 DNA positivity confirmed the diagnosis of DRESS. Other diagnostic tests were carried out to better characterize the extent of the organ involvement and possible complications. Abdominal ultrasound showed splenomegaly in P1 and was normal in P2. Positron emission tomography-computed tomography and cervical lymph node biopsy performed to investigate the relevant lymphoadenomegaly in P2 demonstrated only reactive lymphadenopathy, thus excluding a lymphoproliferative disorder. Anticonvulsants were discontinued in both the patients after the diagnosis and a treatment with methylprednisolone 40 mg daily for 40 days was given to P1; a complete and stable remission of symptoms was observed after a slow tapering of methylprednisolone over a period of 2 months. P2 underwent therapy with prednisone 70 mg daily for 10 days; glucocorticoid was then interrupted for a few days in order to perform lymph nodal biopsy that confirmed the reactive lymphadenopathy: a severe clinical relapse was observed characterized by macular papular rash, systemic lymphadenopathy, itching and asthenia. Prednisone was readministered at a dosage of 50 mg daily for 15 days, reduced to 25 mg and slowly tapered off 5 mg every 2 weeks under strict medical control. Normalization of inflammatory and necrosis markers was observed in P2 after 4 months from the beginning of glucocorticoid therapy; subsequently P2 showed relevant adverse effects: hypertension, headache, acne, hair loss and osteoporosis. In both cases a new anticonvulsant drug was necessary for a relapse of seizures. In P1 clonazepam was introduced after few days of glucocorticoid premedication treatment and was well tolerated in the following months. P2 underwent treatment with levetiracetam for seizure relapse and the drug was well tolerated in the following months. Other drugs were well tolerated after the reaction despite the fear of clinical relapse, such as ibuprofen and clarithromycin in P1; metamizole, tramadol, amoxicillin, clarithromycin, ketoprofen, lercanidipine, and metoclopramide in P2. One month after definitive suspension of glucocorticoids P2 manifested an abrupt increase in blood pressure, dysarthria, confusional state and headache not responsive to treatment. The patient was again hospitalized and the following blood values were observed: Hb 7.8 g/dL, platelets count 11,000 × 10^9^/L, LDH 1560 IU/L, haptoglobin 10 mg/dL, the presence of schistocytes in peripheral blood, total bilirubin 2.2 mg/dL, and ADAMTS13 activity at 7%. After hospital admission the patient manifested purpuric lesions. Brain magnetic resonance imaging was carried out and showed micro hemorrhages. The diagnosis of thrombotic thrombocytopenic purpura (Moskowitz syndrome) was made so treatment with rituximab at dose of 375 mg/m^2^, i.e. once weekly for 4 weeks plus prednisone at 50 mg daily for 2 weeks with tapering after 1 month was instituted. The complete resolution of the clinical picture required a total of 14 months.

### Cases of SJS

Two patients, a 73-year old male (P3) and a 53-year old female (P4) presented SCARs after 4 weeks of therapy with lamotrigine, prescribed to P3 for bipolar disorder with prevailing depressive symptoms in severe cachexia, and phenytoin prescribed to P4 as prophylaxis after acute disseminated encephalomyelitis. Both had developed diffuse maculopapular rash, fever, and 24 h later ulcerations of the oral mucosa, blistering skin lesions, epithelial detachment, conjunctivitis, and diffuse pain. HSV-2 IgM positivity was observed in P3. The clinical pictures and skin biopsy performed in P4 confirmed the diagnosis of SJS (Table 3). Abdominal ultrasound, echocardiogram and chest CT excluded organ involvement. After suspected SJS diagnosis, anticonvulsants were immediately discontinued; both P3 and P4 were treated with hydrocortisone 1 g bolus, followed by prednisone 50 mg/daily for 10 days tapered in the subsequent month. During the hospitalization the patients were treated with intravenous immunoglobulins (IV Ig) 0.4 g/kg daily for 5 days, and with topical agents. Enteral feeding and crystalloid were also required. New anticonvulsants were given: lormetazepam and diazepam in P3 and diazepam in P4. Moreover, P4 developed psychosis as an adverse effect of glucocorticoid therapy and was treated with haloperidol. Echinocandin and teicoplanin were administered for 2 weeks because of prolonged fever and positive blood cultures for *Candida albicans* and methicillin-resistant *Staphylococcus aureus* (MRSA) in P3 and P4, respectively. Both antibiotics were well tolerated by the patients and they were discharged after 3 weeks in good clinical conditions.

### Cases of TEN

Two females, 41 year old (P5), and 21 year old (P6), presented SCARs after 2 weeks of therapy with valproic acid, given to P5 consequently to head trauma, and with lamotrigine, used in P6 for epilepsy. Both patients developed diffuse maculopapular rash and in addition P6 showed severe asthenia and conjunctivitis. Their clinical pictures progressed rapidly to TEN, showing epidermal detachment, mucosal involvement with severe bleeding requiring blood transfusions. In particular, epidermal detachment involved 45 and 95% of the body surface in P5 and P6, respectively. During the hospitalization, P6 developed respiratory distress requiring mechanical ventilation. Diagnostic routine work up on the diagnosis showed leukocytosis and sign of liver damage in both the patients. Infections were detected by urine cultures that showed *Enterococcus faecalis* in P5 and by blood cultures that were positive for *Staphylococcus capitis* and MRSA in P5 and in P6, respectively. Clinical pictures and skin biopsy confirmed the TEN diagnosis (Table 3). Ultrasound abdomen showed hepatomegaly in both patients, and echocardiograms were normal. Based on the diagnosis of TEN, anticonvulsants were stopped and the patients were treated with topical medications, IV Ig at 0.4/kg daily for 5 days and with prednisone 50 mg daily during 2 weeks of the hospitalization with slow tapering with complete remission after 1 month from the discharge. Meropenem and teicoplanin were administered for 2 weeks to treat secondary infections in P5 and P6 respectively and enteral feeding and crystalloid were also required. Antibiotics were perfectly tolerated also after glucocorticoid tapering. In both cases, new anticonvulsant were considered necessary and levetiracetam and diazepam were introduced in P5 and P6 respectively, a few days after the start of cortisone treatment, and were well tolerated in the following months.

## Conclusions

The principal aim of this study was to describe the course of six patients affected by SCARs to anticonvulsants. These drugs, as well as allopurinol, have been already regarded as one of the most common causes of SCARs [19, 20]. The analysis of our clinical data allowed us to determine the tolerance to alternative anticonvulsants and to different drug classes that in the routine practice are often not administered for the fear of a relapse. According to a prospective RegiSCAR study, aromatic AEDs, in particular carbamazepine, phenytoin, and lamotrigine, were considered responsible for the reaction in the 35% of cases. Additional culprit drugs were allopurinol, sulfonamides and other antibiotics involved in another 41% of cases [19]. Two out of 6 SCARs from our study were induced by lamotrigine, three by phenytoin and one by valproic acid. In a recent review 172 cases of DRESS associated with 44 drugs were analyzed: the most frequently implicated was carbamazepine, followed by lamotrigine and phenytoin [21]. In SJS/TEN, an association with 12 “highly suspect” medication was reported that included anticonvulsants, mostly carbamazepine, oxcarbazepine, phenytoin and lamotrigine [20]. These observations confirmed previous clinical data [22, 23]. The reactions in our patients occurred on the first exposure to the drug, with a latency time from 2 to 4 weeks after the beginning of therapy, as already observed [1, 9]. Several potential risk factors for AED hypersensitivity were reported: previous history of AED-induced eruption [24], autoimmune diseases, treatment with corticosteroids, family history of SCARs [25], age below 12 years or above 64 associated with altered drug metabolism [8], head injury, surgery, genetic markers such as HLA-B*15:02 and HLA-A*31:01, and reactivation of HHV-6 and -7, EBV and CMV virus [26, 27].

In our patients we identified several of the above quoted risk factors, in particular: surgery (P1, P2), head trauma (P5), and herpes viral infections in P1, P2 (i.e. HHV-6), and in P3 (i.e. HSV 2); of note, this last patient also suffered from recurrent airway infections due to a cachectic state. All these situations have been reported to potentially determine severe distress and injury to cells that in turn can release internal molecules able to act as damage or danger signals stimulating toll-like receptors on antigen presenting cells activating thus the immune response.

The culprit drugs were identified only by clinical data as up to now no standardized diagnostic test has been adopted in delayed T-cell mediated drug allergy reactions except in contact dermatitis IVa type [28]. Although different skin tests (SPT, ID, patch tests) have been suggested as useful tools for the diagnosis of SCARs [29], more recently drug patch test was reported as the only convenient and safe tool for identifying culprit drugs in DRESS; on the contrary, this procedure was not recommended in SJS/TEN [30, 31]. We did not perform in vivo tests because our patients assumed only AEDs at the onset of SCARs. The clinical course worsened after discontinuation of the culprit drug, as already reported in DRESS [32]. Liver was the extracutaneous organ more frequently affected in our patients, as usually observed in DRESS (70–95%), and in SJS/TEN [33]. In DRESS, hepatic damage was more severe after phenytoin [34], as observed also in our two DRESS patients (P1 and P2). We observed no heart or kidney damage. Signs of renal damage were reported just in the 11% of DRESS patients, mainly, after allopurinol administration [19]. In P1 and P2, a diffuse lymphadenopathy was observed, as already reported in DRESS in nearly 75% of the cases [35]. In particular, in P2 lymph node biopsy showed a benign lymphoid hyperplasia, one of the two histological patterns reported in this syndrome, besides pseudolymphoma [7]. The most common complications we observed were secondary infections i.e. septicemia (4 out of 6 patients; 66%). A transient respiratory failure appeared in one case (P6). P2 developed an autoimmune disease, a sequelae already reported in DRESS especially if not treated with steroids [36]. However, in our patient a thrombotic thrombocytopenic purpura (Moskowitz syndrome) was developed nevertheless a previous high dose steroid therapy followed for several months. The time for complete resolution of the hypersensitivity reaction ranged from 2 to 12 months, compared to a period of 14–345 days reported by others [19, 36].

In respect to the culprit drugs, 5 out of 6 SCARs cases were induced by aromatic anticonvulsant drugs to confirm an increased risk of severe reactions to these molecules in comparison with other AEDs. In two cases (P3 and P6) the culprit drug was lamotrigine, a molecule already associated with a high risk of SJS and TEN [23], through a dose dependent effect [37]. Both lamotrigine and phenytoin, culprit drugs in P3, P4 and P6, were regarded as “highly suspect” compounds associated with SJS/TEN in a pharmacovigilance study [20]. Cross-reactivity between aromatic anticonvulsants ranged from 30 to 58% [38, 39], for this reason in our cases we selected non aromatic drugs, i.e. benzodiazepines and levetiracetam, as an alternative treatment. This choice relied on previous reports suggesting non aromatic AEDs, like gabapentin, as agents with low allergenic potential [39], in particular levetiracetam, was a drug associated with lower rash rates (0–6%) [24].

Some authors advised against treatment with antibiotics or NSAIDs in the DRESS acute period, for unexplained cross-reactivity able to worsen the clinical picture [27]. However, alternative antimicrobials were administered without adverse effects in 16 out of 17 patients affected with antibiotic-related DRESS [40]. Our experience showed that in AED-induced SCARs the risk of hypersensitivity reactions to other drug classes was not increased in comparison with general population. Recently, a strict relationship between SCARs induced by allopurinol and carbamazepine and definite HLA-aplotypes has been identified. This observation might implicate a tight HLA restriction also in adverse reactions to different drugs and consequently a low risk of additional events [41]. In fact, other drugs tolerated were: ibuprofen, clarithromycin, amoxicillin, metamizole, tramadol, ketoprofen, metoclopramide, rituximab, lercanidipine, echinocandin, teicoplanin, haloperidoland meropenem. In our patients, we discontinued the culprit drug and administered steroid therapy at high dosage, a treatment suggested in other studies [42, 43]. High doses of steroid might have an immunosuppressive effect preventing a relapse due to other compounds [41]. The management of our patients differed according to the Unit in which they were admitted: DRESS subjects were treated in our Centre with high doses of steroids, while SJS/TEN subjects were treated in Burn Unit with IVIG, steroids and topic therapy. High doses of IVIG was effective in our SJS/TEN patients, as already reported in some studies [44], although this treatment was considered useless by other Authors [45]. The prognosis was good also as a consequence of careful supportive treatment conducted in specialized Burn Units.

In conclusion, on the basis of our case reports, we can suggest that non aromatic drugs, i.e. benzodiazepines and levetiracetam, are the most safe alternative treatment in SCARs due to the anticonvulsants. Moreover, we observed that drugs belonging to other classes were well tolerated in these patients confirming thus indirectly an HLA restriction for hypersensitivity reactions to other anticonvulsant drugs as well as to carbamazepine and phenytoin.

## Abbreviations

AHS: anticonvulsant hypersensitivity syndrome
AEDs: antiepileptic drugs
SJS: Stevens–Johnson syndrome
TEN: toxic epidermal necrolysis
DRESS: drug reaction with eosinophilia and systemic symptoms
SCARs: severe cutaneous adverse reactions
RegiSCAR: Registry for Serious Cutaneous Reactions
LDH: lactate dehydrogenase
ANA: antinuclear antibodies
HBV: hepatitis virus B
HCV: hepatitis virus C
HSV: herpes simplex virus
HIV: human immunodeficiency virus
EBV: Epstein–Barr virus
CMV: cytomegalovirus
